# Use of rituximab for fulminant and refractory cases of immune checkpoint inhibitor induced myocyte injury clinically presenting with features of myasthenia gravis: a case series

**DOI:** 10.1093/ehjcr/ytaf593

**Published:** 2025-11-17

**Authors:** Imran Naeem Aziz, Rohan Gajjar, Shreyas Nandyal, Omotola Oredipe, Seba Qaddorah, Gedion Yilma Amdetsion, Javier Gomez Valencia, Jonathan Tottleben

**Affiliations:** Department of Internal Medicine, John H Stroger Hospital of Cook County, 1969 W Ogden Avenue, Chicago, IL 60612, USA; Division of Translational Science, Rush University Medical College, Armour Academic Center, 600 S. Paulina St., Suite 403, Chicago, IL 60612, USA; Department of Internal Medicine, John H Stroger Hospital of Cook County, 1969 W Ogden Avenue, Chicago, IL 60612, USA; Department of Cardiology, John H Stroger Hospital of Cook County, 1969 W Ogden Avenue, Chicago, IL 60612, USA; Department of Internal Medicine, John H Stroger Hospital of Cook County, 1969 W Ogden Avenue, Chicago, IL 60612, USA; Department of Internal Medicine, John H Stroger Hospital of Cook County, 1969 W Ogden Avenue, Chicago, IL 60612, USA; Department of Internal Medicine, John H Stroger Hospital of Cook County, 1969 W Ogden Avenue, Chicago, IL 60612, USA; Department of Internal Medicine, John H Stroger Hospital of Cook County, 1969 W Ogden Avenue, Chicago, IL 60612, USA; Department of Cardiology, John H Stroger Hospital of Cook County, 1969 W Ogden Avenue, Chicago, IL 60612, USA; Department of Cardiology, John H Stroger Hospital of Cook County, 1969 W Ogden Avenue, Chicago, IL 60612, USA

**Keywords:** Checkpoint immunotherapy, Immune checkpoint inhibitors immune-related adverse events, Myocarditis, Myositis, Immune checkpoint inhibitor myocarditis, Immunosuppressive therapy, Case series

## Abstract

**Background:**

Immune checkpoint inhibitor-related overlap syndromes are serious and potentially life-threatening complications of immunotherapy. The available evidence is scarce to guide treatment for refractory cases, and patients often experience poorer outcomes.

**Case summary:**

We describe two patients who presented with non-specific symptoms of fatigue and weakness. Both exhibited biomarker evidence of skeletal muscle and myocardial injury, along with clinical features suggestive of myasthenia gravis. The patients rapidly deteriorated, requiring admission to the intensive care unit and eventual intubation due to worsening respiratory status. Despite treatment with high-dose intravenous steroids, there was no significant clinical improvement, although early reductions in biomarker levels were observed. This led to the administration of additional therapies, including intravenous immunoglobulin and plasmapheresis, but these interventions were also ultimately ineffective. All testing for myasthenia gravis returned negative, highlighting how patients with primary myositis or overlap myositis with myocarditis can present with bulbar symptoms that closely mimic those of myasthenia gravis. Following multidisciplinary discussions, rituximab was initiated in both cases, which led to successful weaning from mechanical ventilation and eventual discharge.

**Discussion:**

Our case series highlights how patients with primary myositis or overlap myositis with myocarditis may present with bulbar symptoms that can mimic those of myasthenia gravis. Their clinical course was refractory to initial treatments but improved significantly with rituximab. While current guidelines typically recommend biologic therapies targeting T-cell-mediated immunity, our review of literature found a biological basis for targeting antibody-mediated immunity as well. This approach proved effective, enabling both patients to achieve successful discharge after a prolonged and complex hospitalization, highlighting the importance of considering treatment directed at both T-cell and B-cell immunity.

Learning points

- Immune checkpoint inhibitor overlap syndromes with severe myocyte injury can mimic clinical features of myasthenia gravis, leading to potential diagnostic challenges.
- Current literature and guideline recommendations are largely derived from case reports and small retrospective studies, which lack the robustness to support or refute specific treatment options with certainty.
- Antibody-mediated injury may play a role in immune checkpoint inhibitor myocarditis in addition to T-cell mediated injury (cellular rejection), similar to the pathophysiology of transplant rejection, and should not be entirely overlooked as a potential therapeutic target.

## Introduction

Indications for immune checkpoint inhibitor (ICI) therapies continue to expand across malignancies; however, their use comes at the cost of multisystemic adverse events. Myocarditis is one of the most feared complications of ICI therapy with a reported mortality of 39%–50%^1–3^ in retrospective and pharmacovigilance studies. Patients undergoing combination immune therapy are at a higher risk of developing myocarditis,^4,5^ with reported incidence rates between 0.5% and 1.14%.^2,4,6^

## Summary figure

**Figure:**
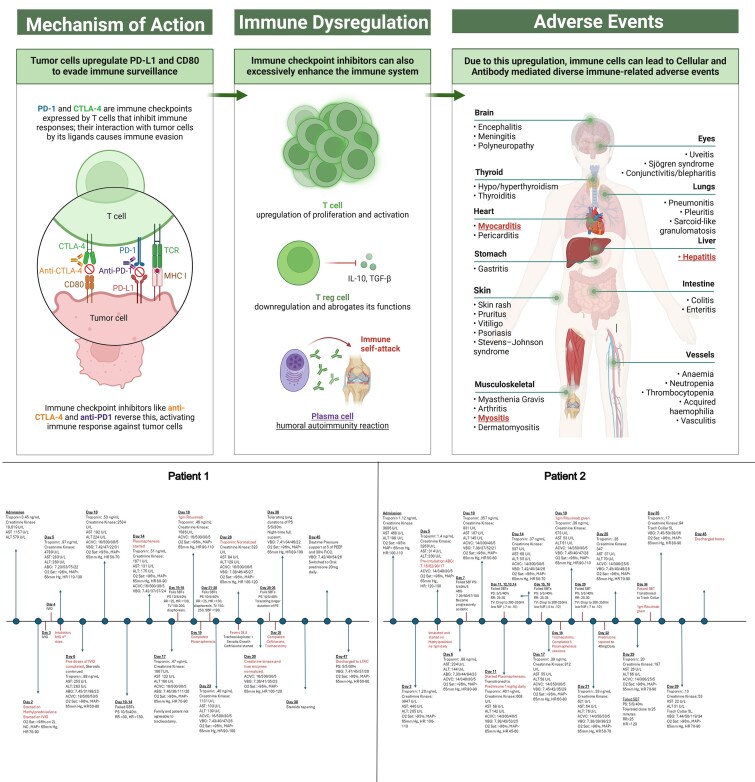
Created in part with BioRender. Aziz, I. (2025) https://BioRender.com/w51w950.

ICI-associated myositis has an overall reported incidence of <1%^7–9^ with a mortality rate ranging from 15% to 24%.^7,8^ However, when myositis occurs concurrently with myocarditis, the mortality rate increases significantly, reaching up to 51%.^7,9^ Such patients are more likely to have prolonged hospitalizations complicated with respiratory failure and ICU admissions.^10^ This has led to growing recognition of a condition known as overlap syndrome, characterized by the concurrent diagnosis of myocarditis, myositis, and myasthenia gravis.

Our case series highlights how patients with primary myositis or overlap myositis with myocarditis may present with bulbar symptoms that can mimic those of myasthenia gravis. In addition, we present a single-centre experience utilizing rituximab for the treatment of refractory myositis and myocarditis.

## Case presentation

### Patient 1

A 68-year-old male with a history of coronary artery bypass grafting and bioprosthetic aortic valve replacement (2018), and renal cell carcinoma (nephrectomy in 2018) developed lung metastases and PET-avid lesions after 4 years of surveillance, leading to the initiation of the first cycle of ipilimumab and nivolumab infusions one month prior to admission.

**Figure 1:**
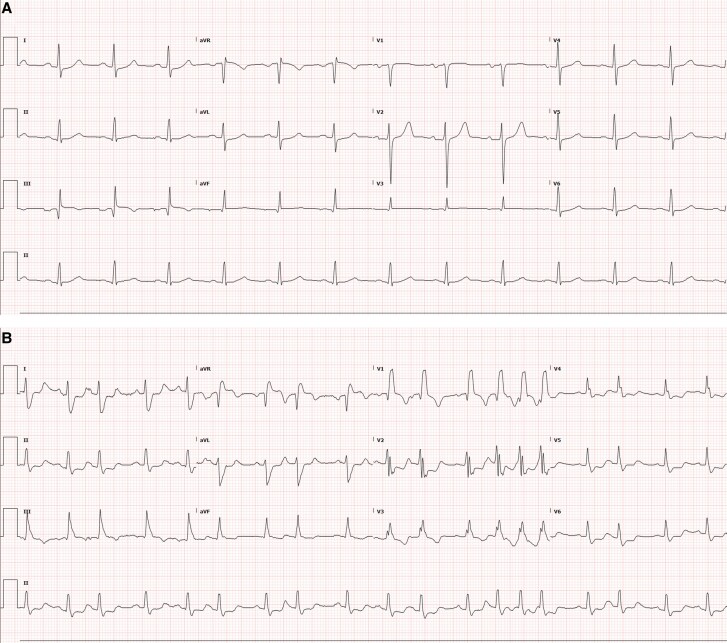
(*A*) Baseline EKG showing normal sinus rhythm. (*B*) Admission EKG showing atrial fibrillation with RBBB and right axis deviation.

He presented to the emergency department (ED) with weakness, palpitations, blurry vision, diffuse arthralgias, and shortness of breath that developed over 5 days. Upon presentation, the patient’s vital signs were temperature 36.4°C (97.5°F), heart rate 88 beats per minute, respiratory rate 18 breaths per minute, blood pressure 156/65 mmHg, and oxygen saturation 97%. Physical examination revealed bilateral ptosis and the use of accessory respiratory muscles. Motor strength was 4/5 in neck flexion and 5/5 in all limbs. The patient’s electrocardiogram (EKG) demonstrated new-onset atrial fibrillation with rate-dependent right bundle branch block (RBBB) and no ischemic changes (*Figure 1*).

Blood analysis revealed acute kidney injury with a BUN of 41 mg/dL and creatinine of 1.4 mg/dL; marked transaminitis (AST 1157 U/L, ALT 579 U/L, LDH 1586 U/L); elevated cardiac biomarkers with troponin I of 3.457 ng/mL and a brain natriuretic peptide level of 167 pg/mL. Initial creatinine kinase (CK) was 19 000 U/L. A CT angiogram confirmed findings of scattered subsegmental atelectasis and demonstrated stable known bilateral pulmonary nodules consistent with the patient's metastatic disease. There was no evidence of pulmonary embolism or thymoma. An arterial blood gas revealed mild respiratory acidosis.

**Figure 2:**
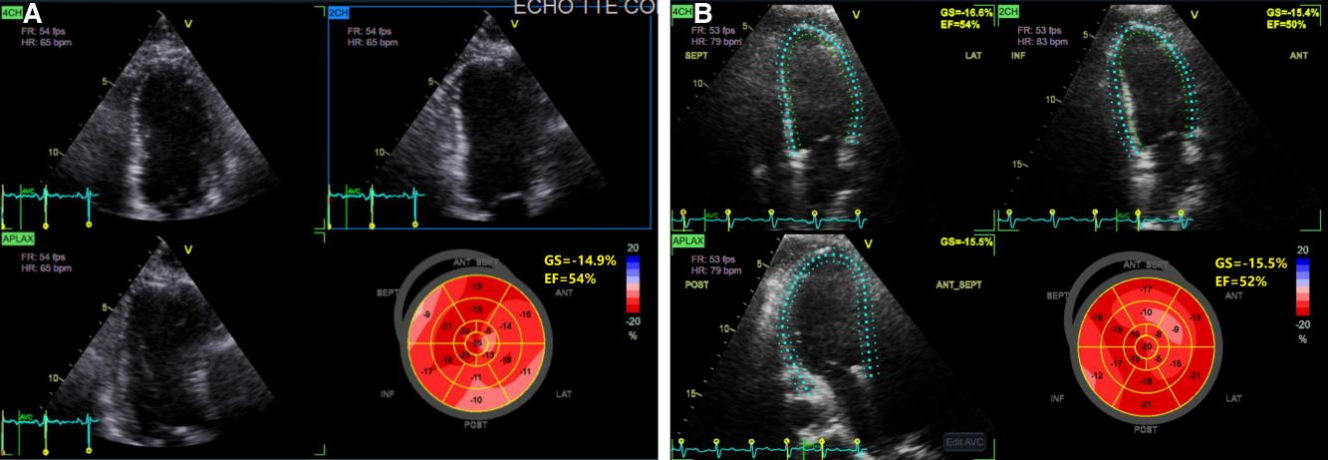
(*A*) The baseline echocardiogram had an estimated ejection fraction reported of 55%–60% with a global longitudinal strain measurement of 14.9%. (*B*) Admission echocardiogram showed an estimated ejection fraction of 55% (in-line video 1) with no regional wall motion abnormalities and a global longitudinal strain of −15.8%.

**Figure 3:**
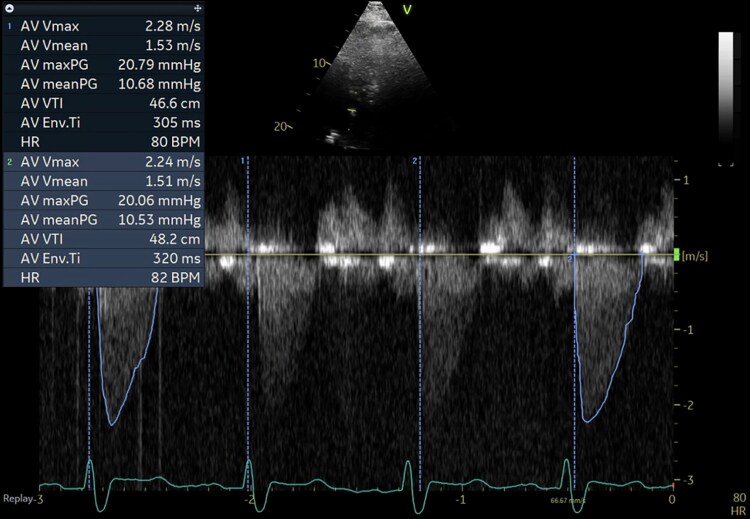
Bioprosthetic aortic valve was functioning normally with a peak systolic velocity of 2.3 m/s, a mean systolic gradient of 11 mm Hg, and an acceleration time of 74 ms.

The patient was admitted to the intensive care unit (ICU) for acute hypoxic and hypercapnic respiratory failure with suspected ICI-mediated myocarditis, myositis, hepatitis, and myasthenia gravis. A transthoracic echocardiogram (TTE) showed an ejection fraction of 60% with no regional wall motion abnormalities (*Figure 2*) and a normally functioning bioprosthetic aortic valve (*Figure 3*). Myasthenia gravis (MG) was suspected, but serological testing for muscle-specific kinase (MuSK) and acetylcholine receptor (AChR) antibodies was negative. Additional testing included a brain magnetic resonance imaging (MRI), which incidentally revealed a pituitary mass measuring 1.5 cm with extension into the suprasellar cistern, which was considered unrelated to the patient’s acute presentation.

Given the severity of multiorgan system involvement, the patient was started on high-dose intravenous methylprednisolone (equivalent to 1 mg/kg of prednisone) and intravenous immunoglobulin (IVIG) at 0.4 g/kg/day. He reverted to normal sinus rhythm from atrial fibrillation and did not require cardioversion during his stay. Despite these interventions, the patient's respiratory status continued to deteriorate, with worsening hypercapnia and hypoxia despite continuous bilevel positive airway pressure support, requiring intubation on the third day of admission. Due to the presence of acute kidney injury, severe liver injury, and the concern for myasthenia gravis, beta-blockers and anticoagulation were withheld. A cardiac MRI was deferred due to acute instability.

By Day 5 of admission, his liver enzymes, troponin, and CK levels had all decreased by more than 50% from their initial levels (*Table 1*). However, despite these biochemical improvements, he failed spontaneous breathing trials, which could not be attributed to preexisting or alternative pulmonary pathology. After multidisciplinary discussions, the patient was started on plasmapheresis (five sessions) and given a dose of rituximab. During his ICU stay, an electromyogram (EMG) showed muscle membrane irritability indicative of inflammatory or necrotizing myopathy, without evidence of a neuromuscular junction disorder. Given these findings, MG was excluded by neurology as a component of the patient’s underlying pathology. Further immune workup, including testing for anti-JO-1, LRP3, anti-PL-7, anti-PL-12, anti-EJ, anti-OJ, anti-SRP, anti-MI-2, anti-MDA-5, anti-TIF-1γ, and anti-NXP-2 antibodies, returned negative. This broad workup helped exclude specific autoimmune myopathies and neuromuscular disorders.

**Table 1:** Patient 1 laboratory data

Test	Reference	Admission value	Fifth day	17th day (1 day pre-rituximab)	47th day (20 days after rituximab)
Haemoglobin, g/dL	11.7–14.5	15.6	11	11.3	8.4
WBC, 10^9^/L	4.4–10.6	10.9	10.1	11.2	7.9
Platelets, 10^9^/L	161–369	178	98	58	156
Sodium, mmol/L	135–145	135	134	136	135
Potassium, mmol/L	3.5–5	4.6	4.1	3.6	3.7
Chloride, mmol/L	100–110	98	105	104	101
Bicarbonate, mmol/L	23–31	26	21	25	26
BUN mg/dL	8–20	41	43	34	27
Creatinine, mg/dL	0.6–1.4	1.4	0.9	0.4	0.4
Calcium, mg/dL	8.5–10.5	9.4	7.5	(−)	(−)
Total protein, gm/dL	6.4–8.3	6.6	6	(−)	(−)
Albumin, g/dL	3.8–5.2	3.7	2.0	(−)	(−)
Total bilirubin, mg/dL	0.2–1.2	1.3	0.4	(−)	(−)
Direct bilirubin, mg/dL	0–0.2	0.3	0.1	(−)	(−)
Alkaline phosphatase, U/L	20–120	79	35	(−)	(−)
GGT, U/L	0–60	34	25	(−)	(−)
AST, U/L	0–40	1157	283	122	(−)
ALT, U/L	5–35	579	260	160	(−)
LDH, U/L	85–210	1586	550	347	(−)
Creatinine kinase, U/L	0–163	19 169	4789	1667	(−)
Troponin-I, ng/mL	0–0.039	3.457	0.975	0.47	(−)
pH	7.32–7.42 (venous^a^)/7.35–7.45 (arterial^b^)	7.28^a^	7.23^b^	7.46^b^	7.47^a^
Carbon dioxide, mm Hg	41–51^a^/32–42^b^	47^a^	53^b^	36^b^	45^a^
Oxygen, mm Hg	27–53^a^/80–100^b^	63^a^	75^b^	111^b^	57^a^
Bicarbonate levels, mmol/L	24–30^a^/20–24^b^	22^a^	22^b^	26^b^	30^a^
Respiratory rate	(−)	(−)	18	16	(−)
Tidal volumes	(−)	(−)	500	500	(−)
FiO_2_%	(−)	(−)	30	30	30
PEEP	(−)	(−)	5	5	5

^a^represents venous sample.

^b^represents an arterial sample.

Following prolonged intubation, the patient and his family decided to proceed with tracheostomy and percutaneous endoscopic gastrostomy tube placement. Ultimately, the patient was weaned from daytime mechanical ventilatory support and was discharged to a long-term acute care facility.

### Patient 2

A 57-year-old woman with recently diagnosed stage IV high-grade endometrial carcinoma presented with dysphagia, dysphonia, and generalized weakness. She had undergone multiple cycles of carboplatin, taxane-based chemotherapy, and pembrolizumab, with her last infusion occurring 5 days prior to presentation.

**Figure 4:**
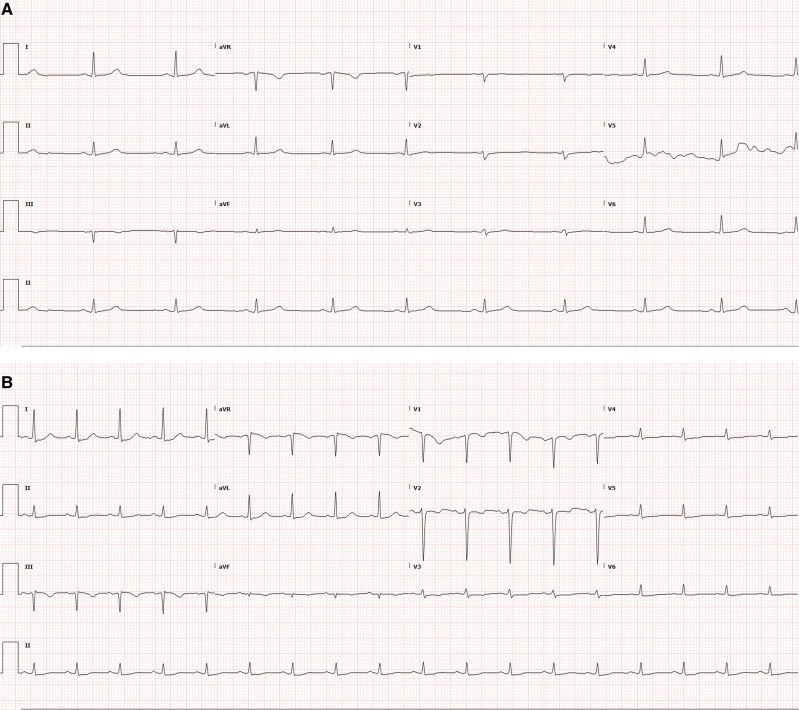
(*A*) Baseline EKG with normal sinus rhythm and low QRS complex voltages in precordial and inferior leads. (*B*) Admission EKG with sinus tachycardia and normal QRS voltage.

On arrival, she was haemodynamically stable with normal vital signs. Physical examination revealed decreased proximal muscle strength in both upper and lower extremities. An EKG showed sinus rhythm without ST segment or T wave abnormalities (*Figure 4*). However, an elevated troponin I level of 1.117 ng/mL (normal, <0.03) was noted, which increased slightly to 1.203 ng/mL 1 h later and plateaued over the next 2 days. Laboratory findings included significant transaminitis (AST 468 U/L, ALT 198 U/L, LDH 1124 U/L) and elevated CK levels (3806 U/L). A computed tomography (CT) angiogram done in the ED was negative for pulmonary embolism and aortic dissection.

**Figure 5:**
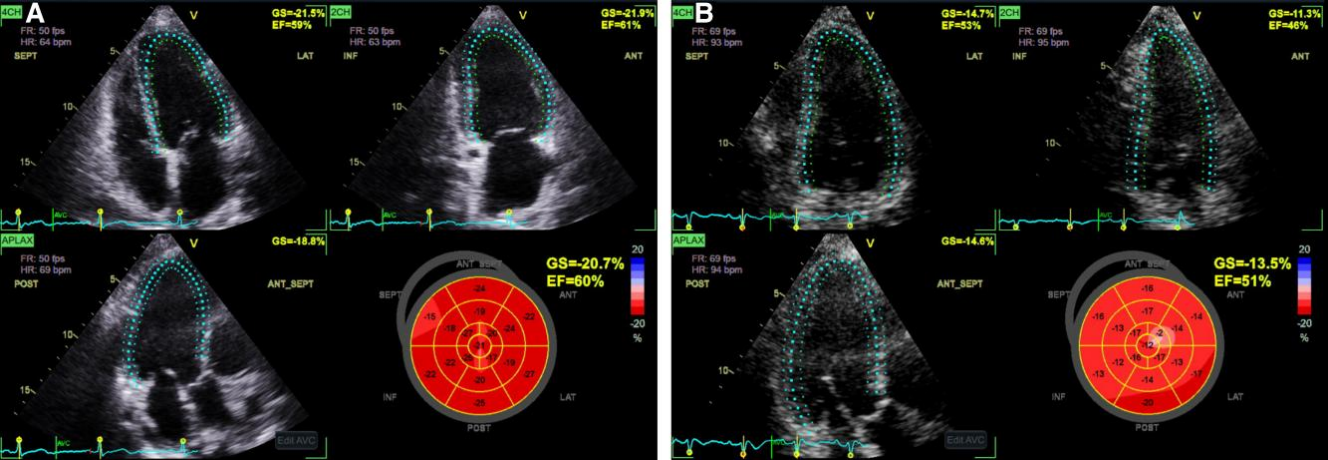
(*A*) Baseline echocardiogram showing an estimated ejection fraction of 60%–65% with a global longitudinal strain of 20.7%. (*B*) Admission echocardiogram with an apparent mild reduction in ejection fraction to 55%–60% (in-line video 2) and a fall in GLS to 13.5%. No regional wall motion abnormalities were noted.

**Figure 6:**
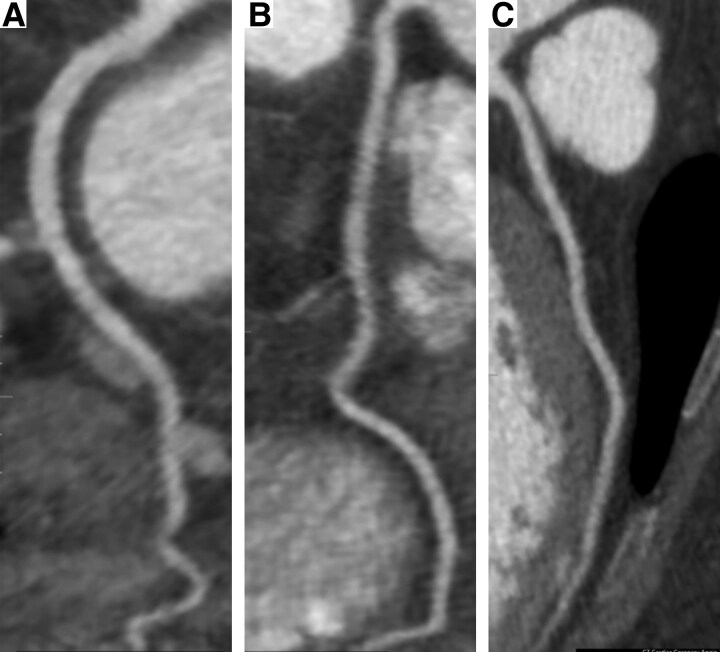
(*A*) Curved multiplanar reformat of Case Y evaluating the left anterior descending coronary artery, (*B*) the left circumflex coronary artery, (*C*) and the right coronary artery. Mild non-obstructive coronary artery disease was demonstrated in the mid-portion of the left circumflex coronary artery, which was unrelated to the patient’s clinical presentation. Mild motion artefact was introduced by the patient’s relatively high heart rate at the time of image acquisition, particularly in the tortuous portions of the vessels, which did not interfere with image interpretation.

On admission, the patient underwent flexible laryngoscopy that disclosed oropharyngeal candidiasis, and she was started on fluconazole. She underwent TTE that demonstrated a preserved left ventricular ejection fraction (55%–60%) but revealed a decline in global longitudinal strain to −13.5%, compared to −20.7% 2 months prior (*Figure 5*). Further evaluation with a CT coronary angiogram revealed no evidence of coronary artery atherosclerotic disease, with a total coronary calcium score of 0 Agatston units (*Figure 6*). In the absence of atherosclerotic disease, ischemic EKG changes, symptoms suggestive of ischaemia, or regional wall motion abnormalities on TTE, and given the concurrent transaminitis and elevated CK levels, the troponin elevation was attributed to ICI myocarditis.

A cardiac MRI was attempted but was not completed due to patient intolerance. Both troponin and CK levels were improving. However, on hospital Day 5, the patient experienced confusion, disorientation, worsening muscle weakness, hoarseness, drooling, fever to 38.5°C (101.3°F), and ptosis. The medical ICU was consulted, and urgent blood work revealed a rise in creatine kinase, hypernatraemia, acute kidney injury, a marked increase in leucocytosis from 12 to 35 k/μL (*Table 2*), and respiratory acidosis. The patient developed acute hypoxic and hypercapnic respiratory failure and required intubation after being transferred to the ICU. She was treated empirically for aspiration pneumonia with broad-spectrum antibiotics.

**Table 2:** Patient 2 laboratory data

Test	Reference	Admission value	Fifth day	10th day	17th day (1 day pre-rituximab)	39th day
Haemoglobin, g/dL	11.7–14.5	11.7	9.3	7.2	9.5	14.4
WBC, 10^9^/L	4.4–10.6	12.1	30.5	17.8	11.6	8.2
Platelets, 10^9^/L	161–369	463	287	145	117	349
Sodium, mmol/L	135–145	135	148	142	139	140
Potassium, mmol/L	3.5–5	4.2	3.5	3.3	3.9	4.9
Chloride, mmol/L	100–110	100	115	107	104	100
Bicarbonate, mmol/L	23–31	28	26	26	31	30
BUN, mg/dL	8–20	25	48	32	19	64
Creatinine, mg/dL	0.6–1.4	0.4	1.7	0.4	0.3	1.0
Calcium, mg/dL	8.5–10.5	8.7	8.3	7.3	(−)	(−)
Total protein, gm/dL	6.4–8.3	5.5	5.4	4.5	(−)	(−)
Albumin, g/dL	3.8–5.2	2.7	2.7	2.3	(−)	(−)
Total bilirubin, mg/dL	0.2–1.2	0.5	0.3	0.3	(−)	(−)
Direct bilirubin, mg/dL	0–0.2	0.2	0.1	0.1	(−)	(−)
Alkaline phosphatase, U/L	20–120	86	162	108	(−)	(−)
GGT, U/L	0–60	8	29	23	(−)	(−)
AST, U/L	0–40	468	314	107	55	22
ALT, U/L	5–35	198	200	145	58	31
LDH, U/L	85–210	1124	809	717	282	(−)
Creatinine kinase, U/L	0–163	3806	3260	831	812	53
Troponin-I, ng/mL	0.039	1.117	1.4	0.357	0.39	0.13
pH	7.32–7.42 (Venous^a^)/7.35–7.45 (Arterial^b^)	(−)	7.16^b^	7.38^a^	7.45^a^	7.44^a^
Carbon dioxide	41–51^a^/32–42^b^	(−)	62^b^	37^a^	42^a^	50^a^
Oxygen	27–53^a^/80–100^b^	(−)	90^b^	52^a^	35^a^	119^a^
Bicarbonate levels	24–30^a^/20–24^b^	(−)	17^b^	21^a^	29^a^	34^a^
Respiratory rate	(−)	(−)	14	14	14	(−)
Tidal volumes	(−)	(−)	340	300	350	(−)
FiO_2_%	(−)	(−)	30	40	30	(−)
PEEP	(−)	(−)	5	5	5	(−)

^a^represents venous sample.

^b^represents an arterial sample.

Neurology was consulted, and an EMG revealed findings suggestive of inflammatory/necrotizing myopathy consistent with myositis, though it provided limited evaluation of post-synaptic neuromuscular junction transmission. The combination of profound proximal-predominant muscle weakness, elevated creatine kinase levels, and EMG findings supported the diagnosis of myositis. The acute onset of bulbar symptoms, including dysphagia, dysphonia, and ptosis, raised concern for new onset MG. The triad of myocarditis, myositis, and myasthenia gravis following pembrolizumab therapy led to a working diagnosis of ‘triple M’ overlap syndrome. Given the acute deterioration, the patient was started on high-dose intravenous steroids (1 g methylprednisolone) for myocarditis and myositis, and pyridostigmine for myasthenia gravis.

Following treatment, fevers resolved, hypernatraemia corrected, CK levels and troponin significantly down trended, and she was stabilized. An MRI of the brain with contrast showed no evidence of acute or chronic abnormalities. However, her clinical status did not improve, and she developed bradycardia attributed to pyridostigmine, leading to its discontinuation. Plasmapheresis was subsequently initiated, and she completed five sessions, but continued to fail spontaneous breathing trials for 7 consecutive days. A repeat EMG showed evidence of muscle membrane irritability with features of inflammatory or necrotizing myopathy and no evidence of a defect in neuromuscular junction transmission. Serological testing for MuSK, AChR, and low-density lipoprotein receptor-related protein 4 came back negative, effectively ruling out MG.

Given her lack of improvement, and after multidisciplinary discussions, the decision was made to administer rituximab. She underwent tracheostomy on the 13th day of mechanical ventilation. The patient was successfully weaned off respiratory support 2 weeks after receiving the first dose of rituximab. After a prolonged hospital stay, she was discharged to an acute rehabilitation facility on a tapered steroid regimen.

As of 10 months post-discharge, she remains actively engaged in physical therapy and continues to make steady progress. A repeat echocardiogram shows a stable ejection fraction and a GLS of 13.3%.

## Discussion

The association between myocarditis and myositis is notably frequent, with a reported prevalence of 25%–40% in various studies.^1–3,6^ Similarly, MG has been observed in ∼10% of myocarditis cases.^1–3^ However, a recent meta-analysis by Nielsen *et al*.^6,10^ indicated a higher co-occurrence rate, reporting MG in 33% of myocarditis cases. Differentiating the underlying aetiology of a patient’s symptoms can be particularly challenging, especially when the presentation includes non-specific symptoms such as fatigue (reported in 80% of cases) and muscle weakness (78%), as well as other manifestations like dyspnoea, chest pain, dizziness, syncope, nausea, fever, and palpitations.^10^ These symptoms often form part of a broader syndrome that can involve myositis, neurotoxicity, and gastrointestinal adverse effects.^10^

Our case series highlights how patients with primary myositis or overlap myositis with myocarditis may present with bulbar symptoms, such as ptosis, dysphonia, and weakness in neck flexion, which are highly suggestive of myasthenia gravis. Aldrich *et al*.^9^ demonstrated that these symptoms occur in up to 20% of cases of myositis alone. In our patients, extensive diagnostic evaluations with serology and EMG were negative for myasthenia gravis. The only definitive findings were evidence of extensive skeletal muscle damage as indicated by markedly elevated CK levels and myocardial injury. The current literature on this confounding clinical presentation is limited to case reports and case series, highlighting the need for greater awareness and further research.^11,12^ Deharo *et al*.^12^ described three cases similar to ours, characterized by features of myositis, myocarditis, and even transaminitis, but with negative testing for MG. However, in contrast to the high mortality rate of 66.6% reported in their series, both of our patients received rituximab and were successfully stabilized and discharged to aftercare.

For refractory cases of ICI myocarditis, the American Society of Clinical Oncology (ASCO) recommends considering agents such as mycophenolate, infliximab, anti-thymocyte globulin, abatacept, or alemtuzumab. However, the evidence supporting these recommendations is limited to case reports for abatacept and alemtuzumab. Support for other medications is obtained from a single-centre retrospective study by Cautela *et al*.^13^ However, the study did not find definitive evidence to support or refute these treatments, except for an increased risk of cardiovascular mortality associated with infliximab. For myositis, both ASCO and the European Society of Medical Oncology (ESMO) recommend high-dose steroids as initial treatment, with plasmapheresis and IVIG reserved for refractory cases. If clinically indicated, the guidelines permit the use of biologic therapy; however, insufficient supportive data prohibit the recommendation of a specific biologic agent. At our centre—a high-volume community safety-net hospital serving a diverse patient population—anti-thymocyte globulin and abatacept were unavailable.

Previous studies have demonstrated that, in addition to T-cell–driven mechanisms, treatment with anti-PD-1 or anti-PD-L1 agents may also influence humoral immunity, potentially enhancing preexisting autoantibodies.^14,15^ T follicular helper (Tfh) and Tfh-like cells express high levels of PD-1, and blockade of this pathway during ICI therapy may contribute to the production of autoreactive antibodies.^16^ Early B cell changes (decline in circulating B cells, increase in a distinct subset of CD21^lo^ B cells and plasmablasts) correlated with the risk for development of severe irAEs following ICI therapy. The changes in B cells preceded the development of clinical complications.^17,18^

These immunological findings are supported by numerous reports in clinical practice where complicated patient presentations attributed to irAE were apparently driven at least in part by known and novel antibody production. Including but not limited to neuromyelitis optica spectrum disorder, inflammatory arthritis, longitudinal extensive transverse myelitis, myositis, and myocarditis.^20^ Balanescu *et al*.^19^ demonstrated complement deposition in endomyocardial biopsy specimens in two of their three patients and referenced animal studies supporting an antibody-mediated process similar to antibody-mediated rejection.

Inan *et al*.^20^ and Schwab *et al*.^21^ described similar cases that showed only partial improvement with high-dose corticosteroids, plasma exchange, and IVIG. A B-cell–mediated mechanism was suspected, and the patients were subsequently treated with rituximab. Both patients demonstrated signs of clinical recovery one month after initiating therapy. The primary distinction from our reported case is that one of the patients had positive anti-titin antibody.^20^ This was not tested for in our patients; however, the clinical features, course of hospitalization, deterioration despite recommended initial treatments, and subsequent recovery after rituximab showed a similar pattern.

In contrast, Fazel *et al*.^22^ described a patient who developed transaminitis, myositis, myocarditis, and myasthenia gravis, which was not treated with rituximab. Despite high-dose steroids, IVIG, and plasma exchange, the patient continued to deteriorate. Interestingly, this clinical decline occurred despite improving biomarkers, including down-trending troponin, transaminases, and CK levels, a pattern similar to what we observed at our centre.

Rituximab depletes B cells via several mechanisms. It can activate the classical complement pathway by binding C1q, leading to membrane attack complex formation and B-cell lysis. It also promotes apoptosis by cross-linking Fc receptors and activating caspase-3.^23^ Additionally, rituximab induces antibody-dependent cell-mediated cytotoxicity by engaging Fcγ receptors on monocytes, macrophages, and NK cells. Unlike newer anti-CD20 agents, rituximab is less associated with direct Fab-mediated cytotoxicity.^23,24^

Our patients were fully ventilator-dependent despite prior treatment with high-dose steroids, plasmapheresis, and IVIG. Rituximab was administered with promising results, leading to noticeable improvements in respiratory status within 7–10 days and eventual weaning from mechanical ventilation after 4 weeks (Patient 1) and 2 weeks (Patient 2), respectively. While the limitations of a descriptive case series in demonstrating the efficacy of rituximab are clear, the growing number of reports and underlying biological plausibility support this as an important hypothesis, one that warrants evaluation in a randomized controlled trial.

## Conclusion

We described two unique cases of ICI-induced myositis and myocarditis overlap syndrome that presented with misleading symptoms suggestive of MG and were successfully treated with Rituximab. These findings suggest that Rituximab may be a therapeutic alternative in these complex cases. However, additional research is required for a better understanding of the underlying mechanisms involved and to confirm the results noted in the present study.

## Lead author biography


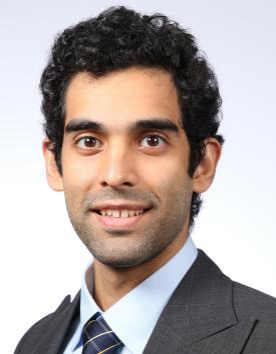


Dr Imran Naeem Aziz recently graduated from residency at Cook County Hospital and is now a first-year Cardiology fellow at Westchester Medical Center. He is also simultaneously pursuing a master’s degree in clinical research from Rush University in Chicago, IL. He has a growing interest in the field of cardio-oncology.

## Supplementary Material

ytaf593_Supplementary_Data

## Data Availability

The data underlying this article will be shared on reasonable request to the corresponding author.
