# Supplementary material for: Use of rituximab for fulminant and refractory cases of immune checkpoint inhibitor induced myocyte injury clinically presenting with features of myasthenia gravis: a case series
Source: Eur Heart J Case Rep. 2025 Nov 17;9(12):ytaf593. doi: 10.1093/ehjcr/ytaf593 (PMC12684965; doi:10.1093/ehjcr/ytaf593)
Supplement: ytaf593_Supplementary_Data [file ytaf593_supplementary_data.zip › Supplement.docx]

**Supplement**

| **Table of Contents** | |
| --- | --- |
| Supplementary References | Page 2 |
| Patient 1 Follow up | Page 3 |
| Patient 2 Follow up | Page 4 |

Supplementary References:

1. Martins F, Sofiya L, Sykiotis GP, Lamine F, Maillard M, Fraga M, et al. Adverse effects of immune-checkpoint inhibitors: epidemiology, management and surveillance. *Nat Rev Clin Oncol.* 2019;16(9):563–580. doi:10.1038/s41571-019-0218-0
2. Rubio-Infante N, Ramírez-Flores YA, Castillo EC, Lozano O, García-Rivas G, Torre-Amione G. Cardiotoxicity associated with immune checkpoint inhibitor therapy: a meta-analysis. *Eur J Heart Fail.* 2021;23(10):1739–1747. doi:10.1002/ejhf.2289
3. Pradhan R, Nautiyal A, Singh S. Diagnosis of immune checkpoint inhibitor-associated myocarditis: A systematic review. *Int J Cardiol*. 2019;296:113-121. doi:10.1016/j.ijcard.2019.07.025
4. Salem JE, Allenbach Y, Vozy A, Brechot N, Johnson DB, Moslehi JJ, et al. Abatacept for Severe Immune Checkpoint Inhibitor-Associated Myocarditis. *N Engl J Med.* 2019;380(24):2377–2379. doi:10.1056/NEJMc1901677
5. Schneider BJ, Naidoo J, Santomasso BD, Lacchetti C, Adkins S, Anadkat M, et al. Management of Immune-Related Adverse Events in Patients Treated With Immune Checkpoint Inhibitor Therapy: ASCO Guideline Update. *J Clin Oncol.* 2021;39(36):4073–4126. doi:10.1200/JCO.21.01440.
6. Haanen JBAG, Carbonnel F, Robert C, Kerr KM, Peters S, Larkin J, et al. Management of toxicities from immunotherapy: ESMO Clinical Practice Guidelines for diagnosis, treatment and follow-up. *Ann Oncol.* 2017;28(suppl_4):iv119–iv142. doi:10.1093/annonc/mdx225
7. Otto F, Seiberl M, Bieler L, Wimmer K, Frey D, Sellner J, et al. Beyond T cell toxicity - Intrathecal chemokine CXCL13 indicating B cell involvement in immune-related adverse events following checkpoint inhibition: A two-case series and literature review. *Eur J Neurol.* 2024;31(7):e16279. doi:10.1111/ene.16279
8. Gatto M, Bjursten S, Jonsson CA, Hedman H, Borg K, Turesson C, et al. Early increase of circulating transitional B cells and autoantibodies to Joint‐Related proteins in patients with metastatic melanoma developing checkpoint Inhibitor–Induced inflammatory arthritis. Arthritis Rheumatol. 2022;75(5):856–863. doi:10.1002/art.42406
9. Charabi S, Engell-Noerregaard L, Nilsson AC, Stenör C. Case report: Longitudinal extensive transverse myelitis with novel autoantibodies following two rounds of pembrolizumab. Frontiers in Neurology. 2021;12. doi:10.3389/fneur.2021.655283.
10. Hirano S, Kojima A, Nakayama Y, Kuroda Y, Kuwabara S, Nakazawa T, et al. A case report of neuromyelitis optica spectrum disorder induced by pembrolizumab treatment for lung adenocarcinoma: a clinical and immunohistochemical study. *BMC Neurol.* 2022;22(1). doi:10.1186/s12883-022-02987-6

**Patient 1 Follow up:**

The patient missed all scheduled follow-up appointments but re-presented from the LTAC three months later for a scheduled brain MRI to evaluate pituitary enlargement identified during his prior admission. The patient was briefly hospitalized due to concerns about care coordination, the need to re-engage multiple specialties, and reassessment of the appropriate discharge destination. He was successfully weaned off nighttime ventilator support and tolerated humidified oxygen via tracheostomy collar.

Repeat MRI demonstrated interval resolution of the previously noted hypoenhancing, mass-like pituitary enlargement, raising the possibility that the initial findings were consistent with ICI-induced hypophysitis. A cardiac MRI was considered; however, it was deferred due to the patient’s inability to perform adequate breath-holding, and the limited utility in management at this time. He was discharged safely to a subacute rehabilitation center with follow-up appointments arranged.

**Patient 2 Follow up:**

Four months after discharge, she passed a tracheostomy capping trial and was subsequently decannulated. Seven months post-discharge, she underwent a total abdominal hysterectomy (TAH), bilateral salpingo-oophorectomy (BSO), and left pelvic lymphadenectomy along with PEG tube removal by the gynecology and general surgery team. Postoperative PET imaging revealed nodal metastatic disease, due to which Paclitaxel and Carboplatin–based regimen was administered under the guidance of Medical Oncology.

As of 10 months post-discharge, she remains actively engaged in physical therapy and continues to make steady progress. A repeat echocardiogram shows a stable ejection fraction and a GLS of 13.3%.
